# The Evolving Role of Vericiguat in Patients With Chronic Heart Failure

**DOI:** 10.7759/cureus.49782

**Published:** 2023-12-01

**Authors:** Ross M Dies, Corrie N Jackson, Chelsi J Flanagan, Evan S Sinnathamby, Noah J Spillers, Pooja Potharaju, Naina Singh, Giustino Varrassi, Shahab Ahmadzadeh, Sahar Shekoohi, Alan D Kaye

**Affiliations:** 1 College of Medicine, Louisiana State University Health Sciences Center, Shreveport, USA; 2 College of Medicine, University of the Incarnate Word School of Osteopathic Medicine, San Antonio, USA; 3 College of Medicine, Louisiana State University Health Sciences Center New Orleans, New Orleans, USA; 4 Department of Anesthesiology, Louisiana State University Health Sciences Center, Shreveport, USA; 5 Department of Pain Medicine, Paolo Procacci Foundation, Rome, ITA

**Keywords:** guideline directed medical therapy, hospitalization, cyclic guanosine monophosphate, mortality, hfref, vericiguat, heart failure, victoria

## Abstract

Heart failure (HF) is a chronic and progressive clinical disorder characterized by an inability to pump sufficient blood to meet metabolic demands. It poses a substantial global healthcare burden, leading to high morbidity, mortality, and economic impact. Current treatments for HF include lifestyle modifications, guideline-directed medical therapies (GDMT), and device interventions, but the need for novel therapeutic approaches remains significant. The introduction of vericiguat, a soluble guanylate cyclase stimulator, has shown promise in improving outcomes for heart failure patients. Vericiguat addresses the underlying pathophysiological mechanisms of heart failure by augmenting the cyclic guanosine monophosphate (cGMP) pathway, leading to enhanced cardiac contractility and vasodilation. Clinical trials evaluating the efficacy and safety of vericiguat, such as the Vericiguat Global Study in Subjects with Heart Failure with Reduced Ejection Fraction (VICTORIA) trial, have demonstrated promising results. It has been shown that vericiguat, when added to standard therapy, reduces the risk of HF hospitalization and cardiovascular death in patients with symptomatic chronic HF with reduced ejection fraction (HFrEF). The addition of vericiguat to the current armamentarium of HF treatments provides clinicians with a novel therapeutic option to further optimize patient outcomes. Its potential benefits extend beyond symptom management, aiming to reduce hospitalizations and mortality rates associated with HF. As with any new treatment, the appropriate patient selection, monitoring, and management of potential adverse effects are essential. Further research is warranted to determine the long-term benefits, optimal dosing strategies, and potential combination therapies involving vericiguat. Its ability to target the cGMP pathway provides a unique mechanism of action, offering potential benefits in improving clinical outcomes for HF patients. Continued investigation and clinical experience will further elucidate the role of vericiguat in the management of HF and its overall impact on reducing the healthcare burden associated with this debilitating condition.

## Introduction and background

Heart failure (HF) has been regarded as an epidemic disease that carries significant morbidity and mortality and reduced quality of life [1,2]. Despite recent advances, high readmission rates for HF exacerbations still challenge healthcare providers [3]. The prevalence of HF worldwide has been estimated to be anywhere from 38 to 65 million individuals as of 2017, with at least six million cases in the United States (US) [3]. The incidence also increases with age [4,5]. The annual economic burden of HF in the US was estimated to be around $31 billion in 2012 and is expected to increase to more than twice this amount by 2030 [4,6]. It is also the leading primary diagnosis for hospitalization in the United States, with around one million discharges every year between 2000 and 2010 [1]. However, the median length of stay has declined in recent years [5].

Heart failure is a clinical syndrome characterized by the reduced ability of the heart to contract and/or accommodate blood, leading to reduced cardiac output and decreased systemic perfusion of the body’s many organs [4,7]. Neurohormonal mechanisms first compensate for initial cardiac dysfunction but eventually prove detrimental and lead to increased ventricular dysfunction followed by failure of adequate circulation [2]. A diagnosis of HF is suggested by several signs and symptoms, which include dyspnea on exertion, reduced exercise tolerance, orthopnea, limb edema, a displaced point of maximal impulse, a third heart sound, elevated jugular venous pressure (JVP), pulmonary rales, and many more. Diagnosis of HF may be further supported by specific findings on echocardiogram, right heart catheterization, chest x-ray, and elevated natriuretic peptides (NP’s), the last of which have a high negative predictive value without being invasive [2,8] Specific etiologies of HF include coronary artery disease (CAD), hypertension, cardiomyopathies, valvular problems, congenital heart defects, arrhythmias, pericardial disease, myocarditis, pulmonary hypertension, and cardiotoxic substances, with the predominant cause in the Western world being CAD [7,8]. 

Heart failure can be classified as related to dysfunction of the left ventricle (LF) and/or right ventricle (RV), with standalone left-sided HF eventually leading to right HF if left untreated [3]. It can also be stratified as acute or chronic, based on the speed of onset; acute HF may be related to cardiogenic shock secondary to acute coronary syndrome, massive pulmonary embolus, or most frequently as an exacerbation of chronic congestive heart failure (CHF) [3,7,9]. The severity of HF is usually portrayed using the New York Heart Association (NYHA) classification, which is also used to indicate prognosis and guide patient management [3]. There are four classes, I-IV, in order of increasing severity [3]. The staged American College of Cardiology/American Heart Association (ACC/AHA) classification complements the NYHA system, and it performs the additional task of identifying those who do not yet have HF but are at risk of developing the condition [7,10].

One of the defining characteristics of HF is the associated ventricular ejection fraction (EF). Heart failure with preserved ejection fraction (HFpEF) is defined as predominately diastolic dysfunction with an EF of >50%, with the main cardiac attributes involving fibrosis and increased ventricular thickness and stiffness [1,3,11]. To date, no treatment modality has been shown to improve morbidity and mortality in HFpEF, and as such, there are few to no evidence-based management recommendations [2,8,12]. Heart failure with reduced ejection fraction (HFrEF) is defined as predominately systolic dysfunction with an EF <40%, and it is marked by progressive LV dilatation and adverse cardiac remodeling [13]. In contrast to HFpEF, HFrEF has a variety of treatment modalities that have been demonstrated to improve outcomes; however, further research is required in this area [2,3,13]. Despite the differences in treatments available, mortality remains high in both, and some data even indicate that the mortality rate may still be higher in HFrEF [11,14].

## Review

Risk factors, complications, and prognosis of heart failure

Ischemic Heart Disease (IHD)

The lasting damage to the myocardium following myocardial infarction (MI) leaves patients susceptible to future myocardial dysfunction, mainly LV dysfunction, and, ultimately, HF. Improvements in the clinical management of MIs have increased post-MI survival [15]. However, studies indicate that improvements in post-MI mortality have increased HF incidence post-MI, especially long-term. In a study of 7,733 patients over 65 hospitalized after their first MI, the Alberta Elderly MI cohort had a decrease in the five-year mortality rate of MI. Among those who survived hospital discharge from their MI event, 76% developed HF within five years, with the majority developing HF within the first year [16].

Hypertension 

Longstanding hypertension has been implicated in various cardiovascular dysfunctions, including HF [17]. The Framingham Study reported an increased incidence of HF related to hypertension, particularly for women [18]. The severity of hypertension coincided with a poorer prognosis for HF as well. Patients who had recorded more severe hypertension (>160/90) regardless of sex, were associated with a double lifetime risk of developing HF compared to subjects with milder hypertension (140/90) [18]. Additionally, several clinical trials have demonstrated the efficacy of antihypertensives to decrease the progression of HF exacerbated by hypertension [17,18].

Diabetes Mellitus (DM)

For several years and across many studies, DM has been an independent risk factor for HF [5,19,20]. Studies have shown that DM patients develop HF at 2.5 times the rate of those who do not have DM [19,20]. In a cross-sectional study comparing matched diabetic and non-diabetic patients, the echocardiogram findings of DM patients noted LV hypertrophy with increased LV wall thickness compared to the non-DM group, suggesting diabetic cardiomyopathy [19]. Additionally, systemic micro- and macrovascular complications of diabetes led to a poorer prognosis for HF patients. Similar to the effects of hypertension, women with diabetes are also more significantly impacted. A prospective cohort study identified nine independent risk factors for HF in postmenopausal women, and diabetes had the strongest risk factor, an adjusted hazard ratio (HR) of 3.1 (95% confidence interval (CI), 2.3 to 4.2) [21].

Obesity and Obesity Paradox

Obesity and increased body mass index (BMI) have been associated with cardiovascular disease development, including HF [5,22,23]. Kenchaiah et al. noted an increase in the incidence of HF amongst both sexes, with a 5% increase in men and a 7% increase in women per one increment increase in BMI within a sample size of 238 men and 258 women [22]. However, once a cardiovascular event has occurred or been established, an interesting phenomenon has been noted in the literature: there is a better prognosis of HF in obese patients than their lean counterparts [23, 24]. In a study conducted with individuals enrolled in a Digitalis Investigational Group Trial and stratified by BMI into underweight, healthy weight, overweight, and obese groups, Curtis et al. reported a decrease in mortality with successive increases in BMI. Most notably, overweight and obese patients had a decreased risk of death, while underweight patients had an increased risk of death (HR=0.88; 95% CI: 0.80-0.96; HR=0.81; 95% CI: 0.720.92; HR=1.21; 95% CI: 0.95-1.53), respectively [25]. Further studies need to be conducted to elucidate this phenomenon.

Minor Risk Factors

Risk factors implicated in a minor increased risk of HF due to current tobacco use in the Coronary Artery Surgery Study is that smoking led to an increase in 47% of the incidence of HF, with a substantial reduction in the risk of cardiovascular disease after just one year of smoking cessation [26]. Additionally, metabolic and electrolyte abnormalities increase the risk of HF, including anemia, dyslipidemia, elevated homocysteine, elevated B-type natriuretic peptide (BNP) levels, chronic kidney disease (CKD) with high creatinine and albuminuria, immune dysfunction with increased proinflammatory cytokines, as well as genetic abnormalities [5,26-28]. Recent meta-analysis data has also implicated HF, leading to increased mortality in COVID-19 infections [29].

Non-modifiable Risk Factors: Age and Sex

Heart failure is a disease often seen in older populations, with the average age of HF patients in the US being 73.2 ± 14 years (Organized Program to Initiate Lifesaving Treatment in Hospitalized Patients with Heart Failure (OPTIMIZE-HF)), with the prevalence of HF increasing with age [30,31]. Certain comorbidities and risk factors can lead to onset among the younger generation. For instance, as mentioned previously, patients with diabetes, on average, were diagnosed with HF 5.5 years earlier than their non-diabetic counterparts [19]. Ozierański et al. conducted a study in Poland comparing patients hospitalized for HF <65 years and ≥ 65 years and their one-year outcomes. Compared to patients within the cohort of ≥65 years, there was an increased prevalence of HFpEF and aortic stenosis, along with concomitant chronic diseases, including hypertension, diabetes, CKD, atherosclerosis, atrial fibrillation (AF), and chronic obstructive pulmonary disease (COPD). Dilated cardiomyopathy was seen in 27.1% of patients with HF hospitalization <65 years of age [27]. The prevalence of HF is relatively similar among men and women [5,18,31,32]. Likewise, significant risk factors such as ischemic heart disease, diabetes, hypertension, and obesity are ubiquitous amongst both sexes; however, there are vast differences in the effects of these risk factors amongst men and women and the type of HF they exhibit. Women have an increased incidence of HFpEF [32]. According to a study conducted by the International Collaboration of Heart Failure Subtypes, male sex was strongly associated with HFrEF [33]. This is thought to be due to the predisposition of HFrEF association with macro-vascular changes such as those seen following MI and HFpEF association with micro-vascular modifications [32].

Clinical Features, Complications, and Prognosis

Heart failure with preserved or reduced ejection fraction often presents signs and symptoms related to fluid overload and redistribution. This includes dyspnea, particularly paroxysmal nocturnal dyspnea, lower extremity edema, and abdominal swelling and fatigue [34]. Physical exam findings, including displaced apex beat, S3 heart sound, and jugular venous distention, are frequently seen in patients with new-onset or chronic and exacerbated HF. This combination of findings can be used to diagnose HF clinically [34]. Following the onset of HF, structural and automatic changes to the myocardium and systemic effects, particularly to the renal system, are due to deleterious compensatory adaptations of the renin-angiotensin-aldosterone system (RAAS) [34,35]. Atrial fibrillation was reported in nearly 1/3 of patients with HF, especially in geriatric populations [34,36]. The pathophysiology of HF, ventricular dysfunction leading to fluid overload, myocardial stretching, hypertrophy, and dysfunction, can precipitate the structural changes causing atrial fibrillation. Likewise, the dysregulated automaticity of the atria can further exacerbate HF [37]. Advanced HF can cause more deleterious ventricular arrhythmias, putting patients at risk of sudden cardiac death and decompensated HF. Some pharmaceutical management of HF, including digoxin and rate-control agents, can cause further ventricular electrical instability [34]. In HF, the reduction in cardiac function leads to hypoperfusion of the kidneys, activating the RAAS system. The aldosterone-stimulated volume retention exacerbates myocardial stretching and increases demands on the already failing heart. In response to myocyte distention, natriuretic peptides are released [35,38]. Additionally, chronic kidney hypoperfusion predisposes patients to CKD and worsens hypertension, diabetic nephropathy, and metabolic derangements, including anemia and uremia, all of which have poor prognostic outcomes [26,38]. With the United States' growing elderly population and increases in medical advancements, it is believed that by 2030, more than eight million people will be diagnosed with HF [39]. Though there has been an improvement in the survival rate within the last 50 years, the estimated annual mortality of HF is 21% in men and 17% in women [26,40]. Likewise, the five-year survival rate after diagnosis of HF is still 50%-60% [28,40]. Hospitalization for exacerbated or decompensated HF is associated with the poorest outcomes, with a 4%-7% in-hospital mortality rate and a 10% mortality rate within 90 days of discharge [41,42].

Current management/treatment of HFrEF

The current standard management of HFrEF involves medications that target the sympathetic nervous system (SNS) and the RAAS, which are the major pathways involved in the pathogenesis of HFrEF [43]. Multiple drug classes are recommended for the treatment of HFrEF, including angiotensin-converting enzyme (ACE) inhibitors, angiotensin receptor blockers (ARBs), beta-blockers, angiotensin receptor neprilysin inhibitors, and diuretics. In addition to pharmacologic therapy, implantable defibrillators and pacemakers for cardiac contractility synchronization are well-documented HFrEF options [44].

Angiotensin-Converting Enzyme Inhibitors

Angiotensin-converting enzyme inhibitors exert their pharmaceutical effects by converting angiotensin I to angiotensin II [45]. Angiotensin II acts as a vasoconstrictor, and when inhibited, it can dilate blood vessels to improve cardiac output and reduce blood pressure [46]. The use of ACE inhibitors in patients with HFrEF is a class I recommendation, according to the ACC/AHA. Heart failure guidelines recommend ACE inhibitors be used to prevent HF in patients with a reduced ejection fraction who also have a history of MI, as the class has demonstrated the ability to reduce morbidity and mortality in this patient population [47]. Although ACE inhibitors are very effective in treating this condition, their use does not come without the possibility of developing adverse effects. Angiotensin-converting enzyme inhibitors are associated with an increased risk of angioedema due to defective degradation of bradykinin and substance P [48]. Other adverse effects include hypotension, renal injury, and hyperkalemia. Nevertheless, this drug class is well tolerated by HFrEF patients, but discontinuation is required in patients experiencing hypotension, worsening kidney function, or hyperkalemia [47]. Angiotensin-converting enzyme inhibitors should be used cautiously in patients with hypotension, renal insufficiency, or hyperkalemia and should not be discontinued abruptly [10].

Angiotensin Receptor Blockers

Angiotensin receptor blockers exert their pharmaceutical effects via the inhibition of the effect of angiotensin II on type 1 angiotensin (AT-1) receptors, inhibiting the effects of angiotensin II on blood vessels. In the Valsartan HF trial in 2001, it was observed that combining ACE inhibitors and ARBs led to worsening kidney function. Thus, these two drug classes are not recommended to be used concomitantly [49]. Angiotensin receptor blockers have a similar adverse effect profile to ACE inhibitors, and their use should be closely monitored within the first two weeks of therapy [43]. Notably, ARBs have a lower risk of angioedema and cough and are a good option for patients who have demonstrated intolerance to ACE inhibitors [47]. Angiotensin receptor blockers have also shown their ability to reduce mortality and hospitalizations due to heart failure exacerbations in various randomized controlled trials [49,50].

Beta-Adrenoceptor Antagonists (Beta-Blockers)

Beta-blockers are another drug class that is the gold standard of therapy in the treatment of HFrEF. Heart failure with reduced ejection fraction is characterized by cardiac beta receptor dysfunction that reduces cardiac inotropic activity [51]. Beta-blockers function by inhibiting the systemic effects of chronic sympathetic activation via the blockade of beta-adrenergic receptors. The three beta-blockers that have been proven effective in reducing the risk of death in patients with HFrEF are bisoprolol, metoprolol succinate, and carvedilol [10,52]. Because of their efficacy in reducing the risk of death in this population, beta-blocker therapy is initiated and maintained in all patients with HFrEF, regardless of whether symptoms improve or not, to reduce the risk of major adverse cardiovascular events [10]. Beta-blockers and ACE inhibitors are complementary drugs, and these two medications are often started simultaneously after a patient has been diagnosed with HFrEF [53].

Angiotensin Receptor-Neprilysin Inhibitors (ARNIs)

An ARNI is a medication composed of an angiotensin receptor blocker and a neprilysin inhibitor. Neprilysin is an enzyme that degrades natriuretic peptides, bradykinin, and other vasoactive peptides [10]. The first United States Food and Drug Administration (FDA)-approved ARNI was sacubitril-valsartan, approved for patients with symptomatic HF. While ARNIs are effective, they are more frequently associated with symptomatic hypotension and increased angioedema incidence than an ACE inhibitor such as enalapril [54]. Angiotensin receptor-neprilysin inhibitors are also recommended to reduce morbidity and mortality in patients with HFrEF. Still, patients must demonstrate an ability to tolerate an ACE inhibitor or an ARB before initiating therapy [55].

Diuretics

Multiple diuretics treat HFrEF to decrease fluid retention and maintain euvolemia [10]. Loop diuretics such as bumetanide, furosemide, and torsemide function by inhibiting sodium or chloride's reabsorption at Henle's loop. Hydrochlorothiazide and other drugs in the thiazide class exert their effects in the distal convoluted tubule, while potassium-sparing diuretics such as spironolactone or eplerenone exert their effects in the collecting duct [56]. Loop diuretics, such as furosemide, are the preferred diuretic agents for HF patients. Thiazide diuretics are helpful for patients with hypertension, HF, and mild fluid retention [10]. While other medications have been shown to reduce morbidity and mortality in HF patients, diuretics' effects on morbidity and mortality are unclear. Therefore, they are not used alone in patients with HF [57,58].

Digoxin

Digoxin has a minimal role in the treatment of HFrEF; the medication is used for symptomatic control only in patients with this condition who are not responding to the other more effective medications [59]. This drug works by inhibiting membrane-bound alpha subunits of the sodium-potassium P-type adenosine triphosphatase (ATPase) pump, inhibiting sodium-calcium exchange, and ultimately increasing the force of myocardial contraction [60, 61]. Digoxin therapy is usually initiated at a low dose because higher doses could potentially harm patients and are not usually required to manage HF. The use of digoxin in HF remains controversial, as two large-scale clinical trials have shown a linear relationship between mortality and digoxin therapy in this patient population [62,63]. Digoxin is best used as adjunctive therapy initiated after treatment regimens, including medications such as ACE inhibitors, ARBs, and beta-blockers, have been optimized [10].

Vericiguat's mechanism of action, pharmacodynamics, and pharmacokinetics

Mechanism of Action

Vericiguat is a direct stimulator of the soluble guanylate cyclase (sGC) enzyme [64]. This enzyme plays a vital role in generating cyclic guanosine monophosphate (cGMP) in cardiac and vascular tissue. Cyclic guanosine monophosphate acts as a messenger in this tissue to mediate cardiac and vasorelaxation via phosphokinase G. Studies have shown that cGMP is deficient in HFrEF and HFpEF [65]. Transforming growth factor-beta produced by monocytes leads to collagen production, which stiffens the heart [66]. Another way to produce cGMP is through the NP particulate guanylate cyclase pathway [64]. This pathway is modified by ARNIs such as sacubitril and valsartan, which are also available for the treatment of HF. Vericiguat and ARNIs could be used together to help treat HF. Vericiguat also stabilizes nitric oxide at its binding site, sensitizing sGC to nitric oxide [67]. It is important to note that, unlike other nitro-vasodilators, vericiguat does not induce tolerance after long-term use [68]. It has been reported that cardiac output increased and systemic vascular resistance decreased at vericiguat doses of 5 mg or higher [69].

Pharmacokinetics and Pharmacodynamics

There is a 93% bioavailability of vericiguat when taken orally with food [67]. Food has been shown to increase the total drug exposure across time of vericiguat [67]. Vericiguat binds primarily to albumin and has a high plasma protein binding percentage (98%) [67]. Sex and body weight are important covariates in vericiguat’s volume of distribution [70]. Vericiguat is metabolized primarily by the liver via uridine diphosphate-glucuronosyltransferase 1A9 and 1A1 [67]. It is formed into an inactive metabolite by these enzymes. Notably, cytochrome P450 (CYP450) is responsible for less than 5% of vericiguat’s metabolism [67]. Vericiguat has an average half-life of 30 hours in patients with heart failure and is cleared at approximately 1.6 liters per hour [67]. It is primarily excreted in the urine as an inactive metabolite and in feces as an unchanged drug [71]. There are also no long-term effects of vericiguat on blood pressure in patients with heart failure and left ventricular EF less than 45% [70]. Age, race, bilirubin, estimated glomerular filtration rate (eGFR), and albumin concentration do not affect the pharmacokinetics of vericiguat [72]. Literature also suggests that female patients of reproductive age should take a pregnancy test before starting vericiguat due to potential hazards to the fetus [67].

Side Effects

Adverse side effects of vericiguat are most notably symptomatic hypotension caused by vasorelaxation, syncope, and anemia [73]. During a previous study, a few patients developed proteinuria, influenza, and pharyngitis, but no severe adverse events or deaths have been recorded. Changes in vasoactive hormones for cGMP, plasma renin activity, noradrenaline, and a decrease in creatinine, urea, and uric acid have also been reported [67].

Conversely, one study did not show a statistically significant difference between vericiguat and placebo regarding worsening renal function. Therefore, the drug may be beneficial in not requiring renal dosing for patients with a mild increase in serum creatinine or potassium levels.

Drug-Drug Interactions

Administration of omeprazole, a protein pump inhibitor (PPI) used in acid reflux treatment, decreased the absorption of vericiguat [67]. However, drug-drug interaction studies indicate that vericiguat is suitable for managing patients with HF and multiple comorbidities requiring multiple medications [71]. If a patient takes a long-acting nitrate, guanylate cyclase stimulator, or phosphodiesterase-5 (PDE5) inhibitor, vericiguat should be avoided, as this can lead to syncope and hypotension [67]. Additionally, patients with severe anemia should avoid vericiguat due to concerns that the drug may decrease hemoglobin levels [73].

Vericiguat use for HFrEF (clinical studies)

Clinical Studies on Vericiguat for the Treatment of HFpEF and HFrEF

There have been several clinical studies regarding the use of vericiguat for HFpEF and HFrEF. The VITALITY-HFpEF study asked if Vericiguat would improve physical limitations on the Kansas City Cardiomyopathy Questionnaire (KCCQ) in patients with HFpEF. Over 700 patients were enrolled in this study. Patients were randomized to receive either 15 mg or 10 mg of vericiguat, or placebo for 24 weeks. Overall, it was found that vericiguat did not increase KCCQ scores in patients with HFpEF [74]. Vericiguat has had very promising results when tested in patients with HF with a reduced ejection fraction. The Vericiguat Global Study in Subjects with Heart Failure with Reduced Ejection Fraction (VICTORIA) Trial was a phase 3 randomized, double-blind, placebo-controlled trial for patients with chronic heart failure. In addition to receiving HF treatment based on guidelines, 5,050 patients were assigned to receive either placebo or vericiguat therapy. Primary outcomes (death from HF or first hospitalization from HF) were recorded for 10.8 months. In the vericiguat group, 897 out of 2,526 primary outcomes were recorded, and 972 out of 2,524 were recorded in the placebo group (HR=0.90; 95% CI: 0.82 to 0.98; P=0.02). There were 691 hospitalizations in the vericiguat group and 747 in the placebo group (HR=0.90; 95% CI: 0.81 to 1.00). There were also 414 deaths from cardiovascular causes in the vericiguat group and 441 deaths from cardiovascular causes in the placebo group (HR=0.93; 95% CI: 0.81 to 1.06). This clinical trial showed that among patients with HFrEF, the incidence of death or hospitalization among patients taking vericiguat was lower than that of those who received a placebo [66].

A meta-analysis by Aimo et al. showed that sodium-glucose cotransporter-2 (SGLT2) inhibitor therapy was not associated with a significantly lower risk of CV death or HF hospitalizations compared to vericiguat, sacubitril, or valsartan. Treatments of sacubitril/valsartan, vericiguat, and SGLT2 inhibitors versus standard-of-care controls were compared. The SGLT2 inhibitor therapy was ranked as the most effective treatment among the three, but this result was not significant [75]. A phase II clinical trial conducted by Gheoghiade et al. was done to determine the optimal dose and tolerability of Vericiguat in patients with HFrEF. There were 456 patients across Europe, North America, and Asia randomized into a placebo or vericiguat dose group. Vericiguat doses were 1.25 mg, 5 mg, or 10 mg, given orally for 12 weeks. N-terminal pro-B-type natriuretic peptide (NT-proBNP) levels were measured throughout. A secondary analysis showed that increasing levels of vericiguat led to an increased reduction of NT-proBNP levels, which was insignificant. There was also a reduction in the rate of any adverse event from 77.2% in the placebo group to 71.4% in the 10 mg vericiguat group [76]. The SOluble guanylate Cyclase stimulatoR in heArT failurE patientS with PRESERVED EF (SOCRATES-PRESERVED) trial was a prospective, randomized, placebo-controlled, double-blind, Phase 2b dose study. The aim was to determine the tolerability and dose regimen of vericiguat in patients with chronic heart failure with preserved EF. Patients received vericiguat once daily at 1.25 mg, 2.5 mg fixed doses, or 5 mg and 10 mg titrated doses from a 2.5 mg starting point for 12 weeks. N-terminal pro-B-type natriuretic peptide and left atrial volume were measured throughout. This study concluded that while vericiguat was well tolerated, there was no change in NT-proBNP or left atrial volume (LAV) at 12 weeks compared with placebo. However, there was an improvement in quality of life in patients with HFpEF [77]. Notably, Ezekowitz et al. demonstrated that vericiguat had the most significant effects on reducing cardiovascular death and HF hospitalization in those with chronic HFrEF when entry-level NT-proBNP was < 8000 pg/ml [78]. A summary of all the studies mentioned above can be found in Table 1.

**Table 1:** Studies on the clinical efficacy of vericiguat HFpEF: heart failure with preserved ejection fraction; KCCQ: Kansas City Cardiomyopathy Questionnaire; HFrEF: heart failure with reduced ejection fraction; CV: cardiovascular; NT-proBNP: N-terminal pro-brain natriuretic peptide; LAV: left atrial volume; SGLT2i: sodium-glucose cotransporter-2 inhibitor; HF: heart failure; eGFR: estimated glomerular filtration rate; WRF: worsening renal function; CVD: cardiovascular death; HFH: heart failure hospitalization

Clinical efficacy of vericiguat			
Author [Year]	Groups studied and intervention	Results and findings	Conclusions
Armstrong et al. [74]	789 patients with HFpEF would be randomized to receive either 15 mg, 10 mg of vericiguat, or a placebo for 24 weeks.	Vericiguat therapy did not increase the KCCQ score	Among patients with HFpEF, 24-week treatment with vericiguat at either 15-mg/d or 10-mg/d dosages compared with placebo did not improve the physical limitation score of the KCCQ.
Armstrong et al. [66]	5,050 patients with HFrEF were randomly assigned to receive either vericiguat therapy (10mg/d) or placebo therapy.	There was a primary outcome event in 35.5% of the vericiguat group and 38.5% of the placebo group. Hospitalization occurred in 27.4% of the vericiguat group and 29% of the placebo group. 16.4% of the vericiguat group died from CV causes, while 17.5% of the placebo group died.	The incidence of death from cardiovascular causes or hospitalization for heart failure was lower among those who received vericiguat than among those who received a placebo.
Gheoghiade et al. [76]	456 patients with HFpEF were randomized to receive either 1.25 mg, 5 mg, or 10 mg of vericiguat, or placebo for 12 weeks	NT-proBNP levels were lower with increasing doses of vericiguat. There was also a reduction in the rate of any adverse event from 77.2% in the placebo to 71.4% in the 10 mg vericiguat group	While vericiguat lowered the NT-proBNP levels, this was not significant.
Pieske et al. [77]	477 patients with HFpEF were randomized to receive either 1.25, 2.5mg fixed doses of vericiguat or 5 or 10 mg of titrated doses of vericiguat starting at 2.5 mg for 12 weeks. NT-proBNP and LAV were assessed at the end of 12 weeks.	Vericiguat was well tolerated but did not significantly change NT-proBNP or LAV amongst patients	Vericiguat does not change NT-proBNP or LAV in patients with HFpEF.
Aimo et al. [75]	Meta-analysis of patients with HFrEF enrolled in phase 3 and phase 2 clinical trials for SGLT2i, sacubitril/valsartan, and vericiguat were analyzed.	There is a trend toward decreased risk of CV death or HF hospitalization with SGLT2i compared to sacubitril/valsartan and Vericiguat therapy. SGLT2i exhibited the greatest effect on HF hospitalization as well as a significant benefit over Vericiguat.	SGLT2i therapy is not associated with a significantly lower risk of CV death or HF hospitalization compared to sacubitril/valsartan or vericiguat. The risk of HF hospitalization does not differ significantly between patients on SGLT2i or sacubitril/valsartan, while SGLT2i therapy is superior to vericiguat.
Voors et al [78]	4,956 patients from the VICTORIA trial had serum creatinine measured at baseline and at weeks 16, 32, and 48. During 48 weeks of treatment, the trajectories in eGFR and creatinine with compared between vericiguat and placebo.	During 48 weeks of treatment, the trajectories in eGFR and creatinine were similar between vericiguat and placebo. WRF, defined as an increase ≥0.3 mg/dL in creatinine from baseline to week 16, was observed in portions of both groups, with no significant difference between groups.	The beneficial effects of vericiguat on the primary outcome of cardiovascular death or hospitalization in those with severe HFrEF are not influenced by eGFR.
Ezekowitz et al. [79]	4,805 patients from the VICTORIA trial with measured NT-proBNP at randomization were evaluated for the primary outcome of CVD or HFH and the secondary outcome of either event on vericiguat vs placebo.	A reduction in the primary composite endpoint and its CVD and HFH components was observed in patients on vericiguat compared with subjects on placebo with NT-proBNP levels up to 8000 pg/ml. Entry-level NT-proBNP >8000 pg/ml had no difference in the primary outcome between vericiguat and placebo.	This provides insight into the potential benefit of vericiguat in high-risk patients with HFrEF, helping identify a patient cohort in whom the medication might be beneficial.

Discussion

Heart failure remains a significant healthcare burden globally, leading to high morbidity, mortality, and economic costs. Despite advancements in treatment, readmission and mortality rates remain high, emphasizing the need for novel therapeutic approaches. Vericiguat, a soluble guanylate cyclase stimulator, has emerged as a promising new agent in pharmacological HF treatment. Clinical trials, including the VICTORIA trial, have demonstrated the efficacy and safety of vericiguat, a newcomer in HF treatment, reducing the risk of HF hospitalization and cardiovascular death in patients with symptomatic chronic HFrEF. Vericiguat's unique mechanism of action, targeting the cGMP pathway, enhances cardiac contractility and vasodilation, addressing some of the underlying pathophysiological mechanisms of HF. The VICTORIA trial, a phase 3 randomized, double-blind, placebo-controlled trial, enrolled over 5,000 patients with chronic HFrEF. The test showed that vericiguat significantly reduced the incidence of death from HF or first hospitalization for HF compared to placebo when added to guideline-directed medical therapy. These findings highlight the potential of vericiguat to improve clinical outcomes and reduce the burden of HF. However, data from the VITALITY-HFpEF trial did not support improvement in the physical limitation score of the KCCQ after treatment with vericiguat.

Current treatments for HF focus on lifestyle modifications, guideline-directed medical therapies, and device interventions. Vericiguat offers a complementary approach, aiming to further optimize patient outcomes by reducing hospitalizations and mortality rates associated with heart failure. By targeting the cGMP pathway, vericiguat enhances the body's natural mechanisms for cardiac contractility and vasodilation, improving cardiac function and relieving symptoms in patients with HFrEF. While vericiguat shows promise, further research is needed to determine the long-term benefits, optimal dosing strategies, and potential combination therapies involving this novel agent. Ongoing studies are evaluating the use of vericiguat in HFpEF, which currently lacks effective treatment options. Exploring the potential benefits of vericiguat in HFpEF could expand its role in managing a broader range of HF patients. Additionally, patient selection, monitoring, and management of potential adverse effects are essential considerations. Vericiguat has been generally well-tolerated, with hypotension being the most common adverse effect. However, close monitoring is necessary to ensure patient safety and identify potential long-term effects.

## Conclusions

In summary, vericiguat represents a promising addition to the current treatment options for HF. Its ability to target the cGMP pathway provides a unique mechanism of action, offering potential benefits in improving clinical outcomes for patients with HFrEF. Continued investigation and clinical experience will further elucidate the role of vericiguat in managing HF and its overall impact on reducing the healthcare burden associated with this debilitating condition. With further research and refinement, vericiguat has the potential to revolutionize the treatment landscape for HF, providing new hope for improved outcomes and quality of life for patients worldwide.
